# Development and Validation of Novel Nomograms to Predict the Overall Survival and Cancer-Specific Survival of Cervical Cancer Patients With Lymph Node Metastasis

**DOI:** 10.3389/fonc.2022.857375

**Published:** 2022-03-17

**Authors:** Jianying Yi, Zhili Liu, Lu Wang, Xingxin Zhang, Lili Pi, Chunlei Zhou, Hong Mu

**Affiliations:** ^1^ Department of Clinical Laboratory, Tianjin First Central Hospital, School of Medicine, Nankai University, Tianjin, China; ^2^ Department of Clinical Laboratory, The Third Central Hospital, Tianjin, China; ^3^ Tianjin Key Laboratory of Extracorporeal Life Support for Critical Diseases, Tianjin, China; ^4^ Artificial Cell Engineering Technology Research Center, Tianjin, China; ^5^ Tianjin Institute of Hepatobiliary Disease, Tianjin, China; ^6^ Department of Gynecology and Obstetrics, Traditional Chinese Medicine Hospital of Xiaoyi City, Xiaoyi, China; ^7^ Department of Clinical Laboratory, People’s Hospital of Xiaoyi City, Xiaoyi, China

**Keywords:** cervical cancer, lymph node metastasis, nomogram, overall survival, cancer-specific survival

## Abstract

**Objective:**

The objective of this study was to establish and validate novel individualized nomograms for predicting the overall survival (OS) and cancer-specific survival (CSS) in cervical cancer patients with lymph node metastasis.

**Methods:**

A total of 2,956 cervical cancer patients diagnosed with lymph node metastasis (American Joint Committee on Cancer, AJCC N stage=N1) between 2000 and 2018 were included in this study. Univariate and multivariate Cox regression models were applied to identify independent prognostic predictors, and the nomograms were established to predict the OS and CSS. The concordance index (C-index), calibration curves, and receiver operating characteristic (ROC) curves were applied to estimate the precision and discriminability of the nomograms. Decision-curve analysis (DCA) was used to assess the clinical utility of the nomograms.

**Results:**

Tumor size, log odds of positive lymph nodes (LODDS), radiotherapy, surgery, T stage, histology, and grade resulted as significant independent predictors both for OS and CSS. The C-index value of the prognostic nomogram for predicting OS was 0.788 (95% CI, 0.762–0.814) and 0.777 (95% CI, 0.758–0.796) in the training and validation cohorts, respectively. Meanwhile, the C-index value of the prognostic nomogram for predicting CSS was 0.792 (95% CI, 0.767–0.817) and 0.781 (95% CI, 0.764–0.798) in the training and validation cohorts, respectively. The calibration curves for the nomograms revealed gratifying consistency between predictions and actual observations for both 3- and 5-year OS and CSS. The 3- and 5-year area under the curves (AUCs) for the nomogram of OS and CSS ranged from 0.781 to 0.828. Finally, the DCA curves emerged as robust positive net benefits across a wide scale of threshold probabilities.

**Conclusion:**

We have successfully constructed nomograms that could predict 3- and 5-year OS and CSS of cervical cancer patients with lymph node metastasis and may assist clinicians in decision-making and personalized treatment planning.

## Introduction

Cervical cancer is a common malignant tumor of the female reproductive system in developing countries and the fourth most commonly diagnosed cancer among women worldwide (1). There were approximately 570,000 newly diagnosed cases and 311,000 deaths from cervical cancer in 2018 (2). Despite the fact that the prevalence and the mortality rate of cervical cancer in developed countries have gradually declined over the past 30 years due to the implementation of human papillomavirus (HPV) vaccination and screening initiatives, cervical cancer is still considered a public health problem, especially among young women in developing countries, where it tends to be aggressive and advanced at the time of diagnosis (3).

American Joint Committee on Cancer (AJCC) and the International Federation of Obstetrics and Gynecology Staging Guidelines (FIGO) are common clinical staging schemes used to evaluate the prognosis of patients with cervical cancer. However, the prediction of prognosis using those staging systems is not sufficiently comprehensive without considering other important personal factors, such as age, race, tumor site, grade, clinical treatments, and lymph node status. Thus, even for patients at the same stage, the survival rate is heterogeneous. In addition, cervical cancer with lymph node metastasis seriously affects the patients’ quality of life, and the prognosis is very poor. In the FIGO stage IB-IIA, the 5-year survival rate of cervical cancer patients with and without lymph node metastasis was 51%–78% and 88%–95%, respectively (4, 5). In 2018, the FIGO made important adjustments classifying cervical cancer with pelvic lymph node metastasis or paraaortic lymph node metastasis as stage IIIC1/2 (6, 7). Therefore, a more comprehensive and personalized prediction model for the prognosis of cervical cancer patients with lymph node metastasis should be developed.

Over the years, nomograms have been utilized to predict the prognosis of various cancers (8, 9). Nomograms can simplify many clinical and demographical factors into a simple visualization evaluation model to predict the probability of events. However, to date, no nomogram has been constructed to predict the prognosis of cervical cancer patients with lymph node metastasis (AJCC N stage=N1). Recent evidence demonstrates that log odds of positive lymph nodes (LODDS) could be used as a parameter for assessing the prognosis of patients according to lymph node metastasis status in various cancers (10–12). However, the prognostic value of LODDS for cervical cancer patients with lymph node metastasis (AJCC N stage=N1) has not yet been investigated.

The purpose of the present study was to identify the factors affecting the prognosis of cervical cancer patients with lymph node metastasis and establish nomogram models based on LODDS to predict the OS and CSS for those patients.

## Materials and Methods

### Study Population and Selection Criteria

**Figure 1:**
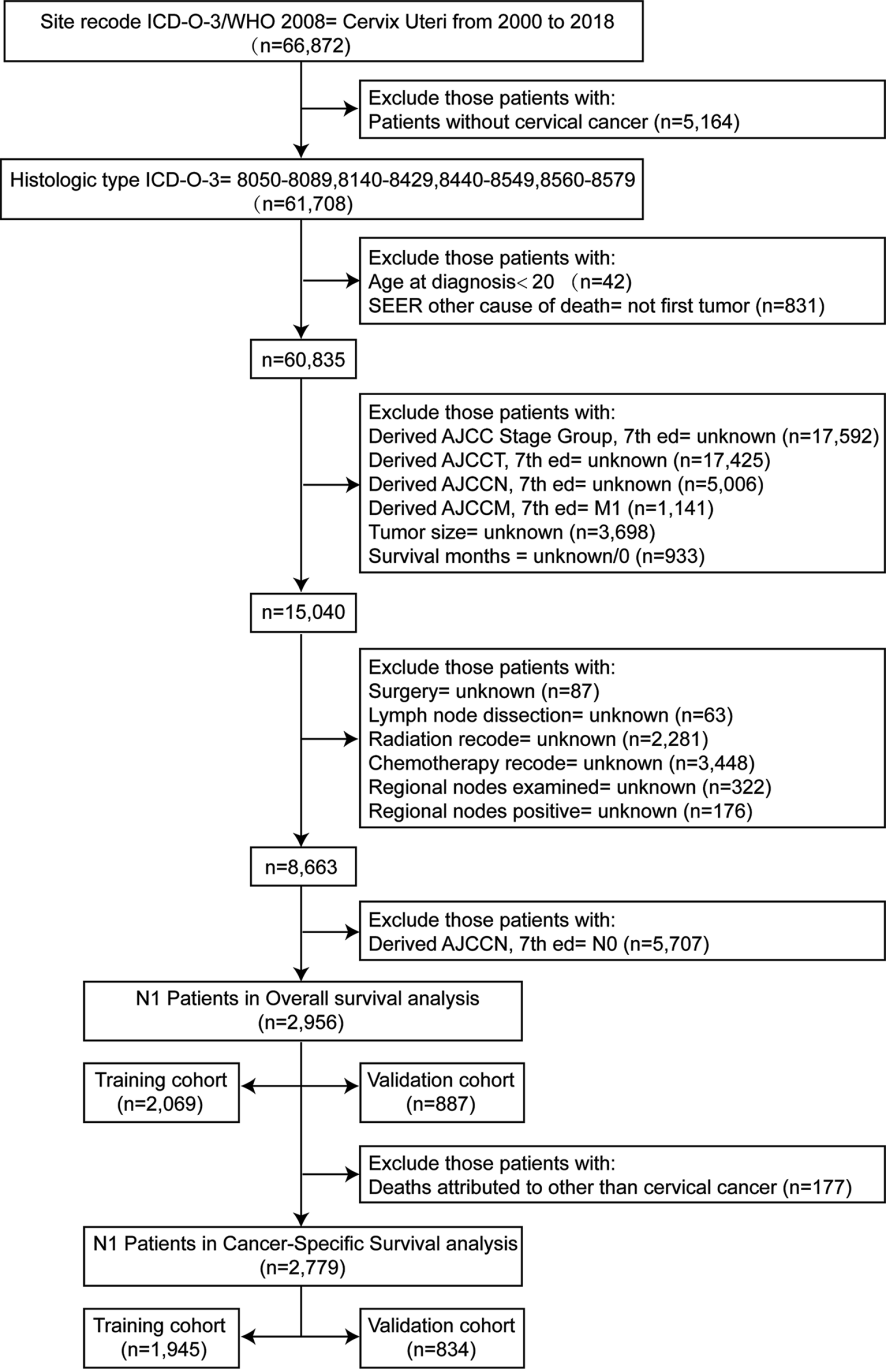
The flow chart of the patient’s selection process.

This retrospective study collected and analyzed the clinicopathological characteristics of patients with cervical cancer diagnosed between 2000 and 2018 from the SEER database, with the accession number 13738-Nov2020. The SEER database is the largest population-based tumor registry system in the United States (13). Medical ethics statement or approval review was not required for this study since all de-identified data were made publicly available. Patients who met the following criteria were included: (1) site recode ICD-O-3/WHO2008=Cervix Uteri; (2) patients diagnosed with cervical cancer [histologic type ICD-O-3 = 8050-8089 (squamous cell carcinoma), 8140-8429 (adenocarcinoma), 8440-8549 (adenocarcinoma), 8560-8579 (adenosquamous carcinoma)] from 2000 to 2018; (3) AJCC N stage=N1; (4) cervical cancer was the only primary malignancy; and (5) demographic variables and tumor characteristics were procurable. Patients with unknown TNM stage records, incomplete tumor grade records, missing survival time, no information concerning treatment, and those with distant metastasis were excluded. Eventually, 2,956 and 2,779 cervical cancer patients with lymph node metastasis were in the cohort. All eligible patients were randomly divided into the training cohort (2,069 and 1,945 cases) and the validation cohort (887 and 834 cases) at a ratio of approximately 7:3 for OS and CSS, respectively. A detailed flow diagram of the patient’s selection process is shown in **Figure 1**.

### Data Collection

Data, including age, race, tumor site, tumor size, LODDS, radiotherapy, chemotherapy, lymph node dissection, surgery, T stage, histology, and grade, were collected for each patient. LODDS was formulated by log ([the amount of positive lymph nodes + 0.5]/[the amount of harvested lymph nodes - the amount of positive lymph nodes + 0.5]) (14).

OS and CSS were the primary endpoints. OS was calculated from the date of diagnosis to the date of death due to any cause. CSS was calculated from the date of diagnosis to the date of death caused by cervical cancer. The optimal cutoff value of tumor size and LODDS was analyzed using the X-tile software (Version 3.6.1, Yale University School of Medicine, USA).

### Statistical Analysis

Variables with *P* value < 0.05 in the univariate Cox regression model were incorporated in the multivariate Cox regression model to identify the independent prognostic factors associated with OS and CSS, and to estimate the hazard ratios and 95% confidence intervals. The prognostic nomograms were built based on the results of the multivariate Cox proportional hazards regression analysis, which was used to predict the 3- and 5-year OS and CSS by representing the sum of points for each factor. The C-index and the AUC of the ROC curve were calculated to evaluate the accuracy values of the prognostic models. Then, the calibration curves were used to assess the relationship between the predicted probabilities and actual outcomes, and the calibration was evaluated by bootstrapping 1,000 times. Additionally, DCA was applied to estimate the clinical utility of the established nomograms by quantifying the net benefits at numerous threshold probabilities. All statistical analyses and plots were carried out with SPSS 25.0 and R software (version 4.1.0). *P* value < 0.05 was considered as statistically significant.

## Results

### Baseline Clinicopathological Features of Patients

**Figure 2:**
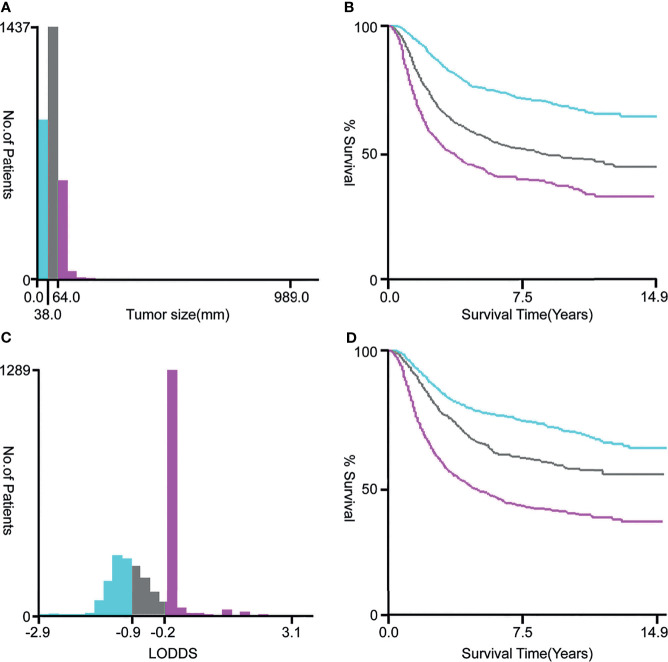
The optimal cutoff values for tumor size **(A, B)** and LODDS **(C, D)** *via* X-tile software analysis. The optimal tumor size cutoff values calculated by overall survival were 38 and 64 mm. The optimal LODDS cutoff values calculated by overall survival were -0.9 and -0.2. Tumor size was divided into ≤ 38 mm (sky blue), 39–64 mm (gray), and ≥ 65 mm (pink purple) subgroups. The LODDS was divided into three subgroups: LODDS1 (LODDS ≤ -0.9, sky blue), LODDS2 (-0.9 < LODDS ≤ -0.2, gray), and LODDS3 (LODDS > -0.2, pink purple). LODDS, log odds of positive lymph nodes.

As shown in **Figure 1**, after a rigorous screening estimation, 2,956 cervical cancer patients diagnosed with lymph node metastasis between 2000 and 2018 were included in the cohort to explore the prognostic factors for OS. All eligible patients were randomly divided into the training cohort (2,069 cases) and the validation cohort (887 cases) at a ratio of approximately 7:3. According to the optimal cutoff value by the X-tile software, the tumor size was divided into ≤ 3.8, 3.9–6.4, and ≥ 6.5 cm subgroups (**Figures 2A, B**). LODDS was then divided into three subgroups: LODDS1 (LODDS ≤ -0.9), LODDS2 (-0.9 < LODDS ≤ -0.2), and LODDS3 (LODDS > -0.2) (**Figures 2C, D**). The patients’ detailed clinicopathologic features are summarized in **Table 1**.

**Table 1:** Baseline clinicopathological features of cervical cancer patients with lymph node metastasis in the training cohort and the validation cohort.

Variables	Training cohort	Validation cohort	P value
	(N = 2069)	(N = 887)	
Age(year)			0.246
20-59	1566 (75.7%)	653 (73.6%)	
≥60	503 (24.3%)	234 (26.4%)	
Race			0.091
Black	153 (7.4%)	47 (5.3%)	
White	1639 (79.2%)	711 (80.2%)	
Other	277 (13.4%)	129 (14.5%)	
Tumor site			0.892
C53.0-endocervix	430 (20.8%)	177 (20.0%)	
C53.1-exocervix	53 (2.6%)	26 (2.9%)	
C53.8-overlapping lesion	40 (1.9%)	18 (2.0%)	
C53.9-cervix uteri	1546 (74.7%)	666 (75.1%)	
Tumor size(mm)			0.164
≤38	669 (32.3%)	260 (29.3%)	
39-64	832 (40.2%)	358 (40.4%)	
≥65	568 (27.5%)	269 (30.3%)	
LODDS			0.233
LODDS1	716 (34.6%)	281 (31.7%)	
LODDS2	501 (24.2%)	214 (24.1%)	
LODDS3	852 (41.2%)	392 (44.2%)	
Radiotherapy			0.712
No	168 (8.1%)	68 (7.7%)	
Yes	1901 (91.9%)	819 (92.3%)	
Chemotherapy			0.331
No	197 (9.5%)	74 (8.3%)	
Yes	1872 (90.5%)	813 (91.7%)	
Lymph node dissection			0.207
No	839 (40.6%)	382 (43.1%)	
Yes	1230 (59.4%)	505 (56.9%)	
Surgery			0.415
Preserve uterus	833 (40.3%)	377 (42.5%)	
Hysterectomy	1236 (59.7%)	510 (57.5%)	
T stage			0.481
T1	898 (43.4%)	361 (40.7%)	
T2	707 (34.2%)	308 (34.7%)	
T3	375 (18.1%)	178 (20.1%)	
T4	89 (4.3%)	40 (4.5%)	
Histology			0.178
Adenocarcinoma	425 (20.5%)	158 (17.8%)	
Adenosquamous carcinoma	124 (6.0%)	61 (6.9%)	
Squamous cell carcinoma	1520 (73.5%)	668 (75.3%)	
Grade			0.145
I	145 (7.0%)	45 (5.1%)	
II	806 (39.0%)	336 (37.9%)	
III	1009 (48.8%)	451 (50.8%)	
IV	109 (5.3%)	55 (6.2%)	

LODDS, log odds of positive lymph nodes.

In the training and validation cohorts, there were 75.7% and 73.6% of the patients between 20 and 59 of age, respectively. The majority of the patients were white [in the training cohort (79.2%) and the validation cohort (80.2%)]. The tumor site, histology, and grade were predominantly classified as confined to the cervix uteri (74.7%, 75.1%), squamous cell carcinoma (73.5%, 75.3%), and III (48.8%, 50.8%) in either the training or validation cohort, respectively. Regarding therapy, 91.9% and 92.3% of patients received radiotherapy, 90.5% and 91.7% received chemotherapy, while 59.7% and 57.5% underwent hysterectomy in the training and validation cohorts, respectively. The chi-square test indicated no evident differences between the training and validation cohorts (all *P* > 0.05).

After excluding patients whose deaths were caused by conditions other than cervical cancer, 2,779 cervical cancer patients with lymph node metastasis were included in the cohort to explore the prognostic factors for CSS. Similarly, all eligible patients were randomly divided into the training cohort (1,945 cases) and the validation cohort (834 cases) at a ratio of approximately 7:3.

### Cox Regression Analyses to Identify Prognostic Factors for OS and CSS

Univariate and multivariate Cox proportional hazard regression analyses were applied to investigate the prognostic factors for OS and CSS. The results of the univariate Cox analysis indicated that tumor size, LODDS, radiotherapy, surgery, T stage, histology, and grade were significantly (*P* < 0.05) associated with OS (**Table 2**) and CSS (**Table 3**). Based on the elements identified by univariate Cox analysis, multivariate Cox analyses of OS and CSS were performed. Tumor size, LODDS, radiotherapy, surgery, T stage, histology, and grade were all independent prognostic factors for OS (**Table 2**). Independent prognostic factors for CSS were the same as those for OS (**Table 3**).

**Table 2:** Univariate and multivariate Cox regression analysis of OS in cervical cancer patients with lymph node metastasis (training cohort).

Variables	Univariate Analysis		Multivariate Analysis	
	HR (95%CI)	P value	HR (95%CI)	P value
Age(year)				
20-59	Reference			
≥60	1.059 (0.933, 1.202)	0.375		
Race				
Black	Reference			
White	1.068 (0.858, 1.330)	0.555		
Other	1.113 (0.860, 1.441)	0.416		
Tumor site				
C53.0-endocervix	Reference			
C53.1-exocervix	1.226 (0.867, 1.733)	0.249		
C53.8-overlapping lesion	1.085 (0.743, 1.587)	0.672		
C53.9-cervix uteri	0.987 (0.861, 1.131)	0.853		
Tumor size(mm)				
≤ 38	Reference		Reference	
39-64	1.391 (1.169, 1.655)	0.014	1.408 (1.185, 1.672)	<0.001
≥ 65	1.870 (1.544, 2.266)	0.008	1.912 (1.582, 2.311)	<0.001
LODDS				
LODDS1	Reference		Reference	
LODDS2	1.225 (1.029, 1.459)	0.023	1.237 (1.039, 1.473)	<0.001
LODDS3	1.833 (1.573, 2.136)	0.007	1.825 (1.567, 2.126)	<0.001
Radiotherapy				
No	Reference		Reference	
Yes	0.611 (0.476, 0.785)	0.006	0.644 (0.523, 0.793)	<0.001
Chemotherapy				
No	Reference			
Yes	1.090 (0.832, 1.427)	0.532		
Lymph node dissection				
No	Reference			
Yes	0.861 (0.731, 1.014)	0.073		
Surgery				
Preserve uterus	Reference		Reference	
Hysterectomy	0.728 (0.616, 0.859)	0.005	0.664 (0.580, 0.760)	<0.001
T stage				
T1	Reference		Reference	
T2	1.314 (1.136, 1.519)	0.007	1.332 (1.153, 1.539)	<0.001
T3	1.729 (1.459, 2.048)	0.010	1.784 (1.510, 2.108)	<0.001
T4	2.417 (1.853, 3.153)	0.002	2.497 (1.920, 3.249)	<0.001
Histology				
Adenocarcinoma	Reference		Reference	
Adenosquamous carcinoma	1.748 (1.414, 2.161)	0.003	1.743 (1.412, 2.152)	<0.001
Squamous cell carcinoma	0.706 (0.613, 0.813)	0.015	0.708 (0.615, 0.814)	<0.001
Grade				
I	Reference		Reference	
II	2.094 (1.430, 3.068)	0.002	2.096 (1.431, 3.070)	<0.001
III	3.037 (2.084, 4.427)	0.010	3.025 (2.076, 4.409)	<0.001
IV	4.952 (3.282, 7.474)	0.006	4.931 (3.269, 7.438)	<0.001

OS, overall survival; LODDS, log odds of positive lymph nodes.

**Table 3:** Univariate and multivariate Cox regression analysis of CSS in cervical cancer patients with lymph node metastasis (training cohort).

Variables	Univariate Analysis		Multivariate Analysis	
	HR (95%CI)	P value	HR (95%CI)	P value
Age(year)				
20-59	Reference			
≥60	1.052 (0.917, 1.207)	0.469		
Race				
Black	Reference			
White	1.103 (0.868, 1.400)	0.423		
Other	1.143 (0.862, 1.516)	0.352		
Tumor site				
C53.0-endocervix	Reference			
C53.1-exocervix	1.237 (0.859, 1.781)	0.252		
C53.8-overlapping lesion	1.083 (0.714, 1.642)	0.708		
C53.9-cervix uteri	0.998 (0.732, 1.360)	0.996		
Tumor size(mm)				
≤38	Reference		Reference	
39-64	1.443 (1.193, 1.745)	0.003	1.448 (1.199, 1.748)	<0.001
≥65	2.005 (1.628, 2.469)	0.005	2.029 (1.652, 2.493)	<0.001
LODDS				
LODDS1	Reference		Reference	
LODDS2	1.279 (1.057, 1.549)	0.012	1.291 (1.066, 1.563)	<0.001
LODDS3	1.969 (1.666, 2.328)	0.008	1.974 (1.671, 2.332)	<0.001
Radiotherapy				
No	Reference		Reference	
Yes	0.562 (0.430, 0.734)	0.001	0.617 (0.495, 0.770)	<0.001
Chemotherapy				
No	Reference			
Yes	1.202 (0.892, 1.620)	0.226		
Lymph node dissection				
No	Reference			
Yes	0.915 (0.767, 1.092)	0.326		
Surgery				
Preserve uterus	Reference		Reference	
Hysterectomy	0.712 (0.596, 0.851)	0.002	0.670 (0.580, 0.775)	<0.001
T stage				
T1	Reference		Reference	
T2	1.306 (1.115, 1.528)	0.001	1.321 (1.130, 1.544)	<0.001
T3	1.751 (1.460, 2.100)	0.007	1.790 (1.497, 2.141)	<0.001
T4	2.548 (1.931, 3.362)	0.005	2.612 (1.986, 3.435)	<0.001
Histology				
Adenocarcinoma	Reference		Reference	
Adenosquamous carcinoma	1.771 (1.416, 2.215)	0.004	1.772 (1.419, 2.212)	<0.001
Squamous cell carcinoma	0.671 (0.578, 0.779)	0.013	0.671 (0.578, 0.779)	<0.001
Grade				
I	Reference		Reference	
II	1.899 (1.276, 2.827)	0.002	1.894 (1.273, 2.818)	<0.001
III	2.894 (1.958, 4.279)	0.011	2.893 (1.957, 4.276)	<0.001
IV	4.743 (3.096, 7.264)	0.006	4.734 (3.092, 7.246)	<0.001

CSS, cancer-specific survival; LODDS, log odds of positive lymph nodes.

### Construction of Prognostic Nomograms

**Figure 3:**
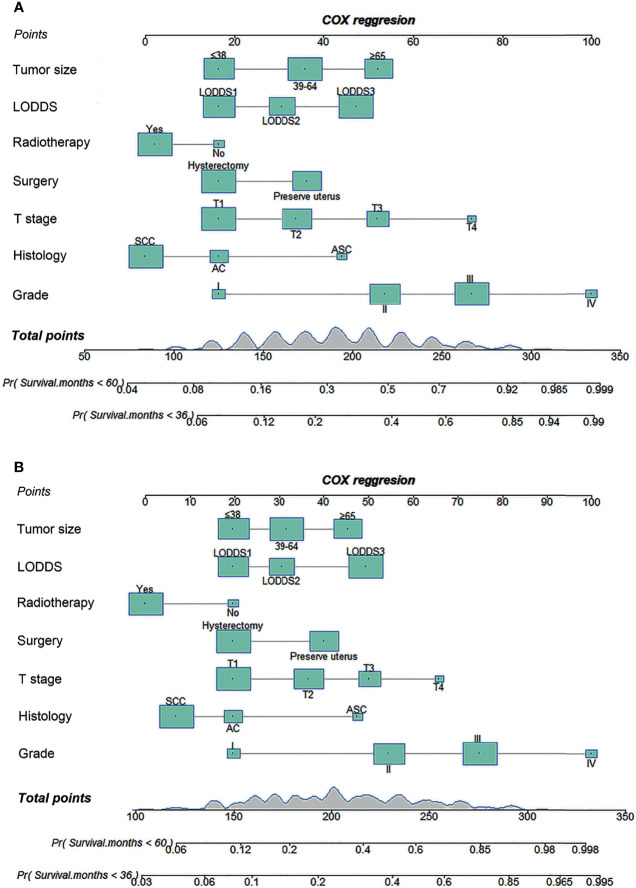
Nomograms for predicting 3- and 5-year OS **(A)** and CSS **(B)** in cervical cancer patients with lymph node metastasis. OS, overall survival; CSS, cancer-specific survival; LODDS, log odds of positive lymph nodes; SCC, squamous cell carcinoma; AC, adenocarcinoma; ASC, adenosquamous carcinoma.

Seven factors (tumor size, LODDS, radiotherapy, surgery, T stage, histology, and grade) were selected for developing nomograms to predict 3- and 5-year survival (**Figure 3**). In those nomograms, each predictor was given a score on the scale by its corresponding point; the total score was calculated by adding the scores of each predictor. Then the 3- and 5-year survival were evaluated by drawing a vertical line from the total score to the corresponding survival axes on those nomograms. As revealed in the nomogram for OS, the grade was the most influential factor, followed by the T stage and the tumor size (**Figure 3A**). Besides, the tumor grade had the largest contribution to the prognosis in the CSS nomogram, followed by the T stage and LODDS (**Figure 3B**).

### Validation and Clinical Value of Prognostic Nomograms

**Figure 4:**
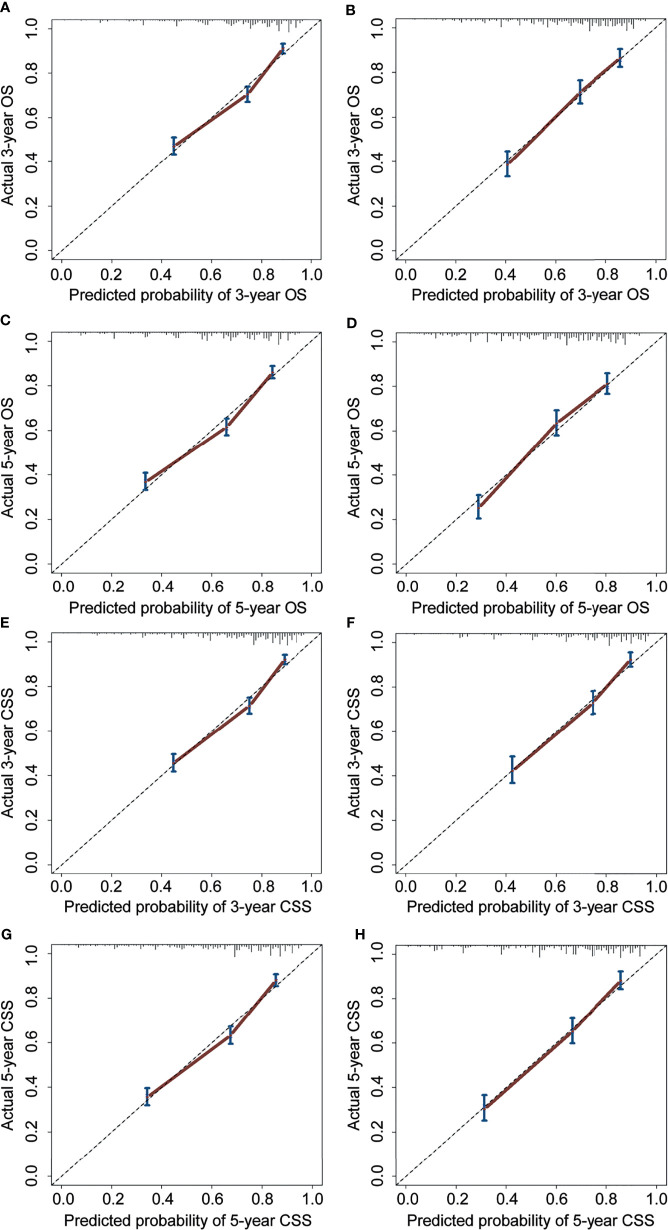
Calibration curves for 3- and 5-year OS and CSS of the prognostic nomograms. Calibration curves for 3- and 5-year OS prediction in the training cohort **(A, C)** and the validation cohort **(B, D)**. Calibration curves for 3- and 5-year CSS prediction in the training cohort **(E, G)** and the validation cohort **(F, H)**. OS, overall survival; CSS, cancer-specific survival.

**Figure 5:**
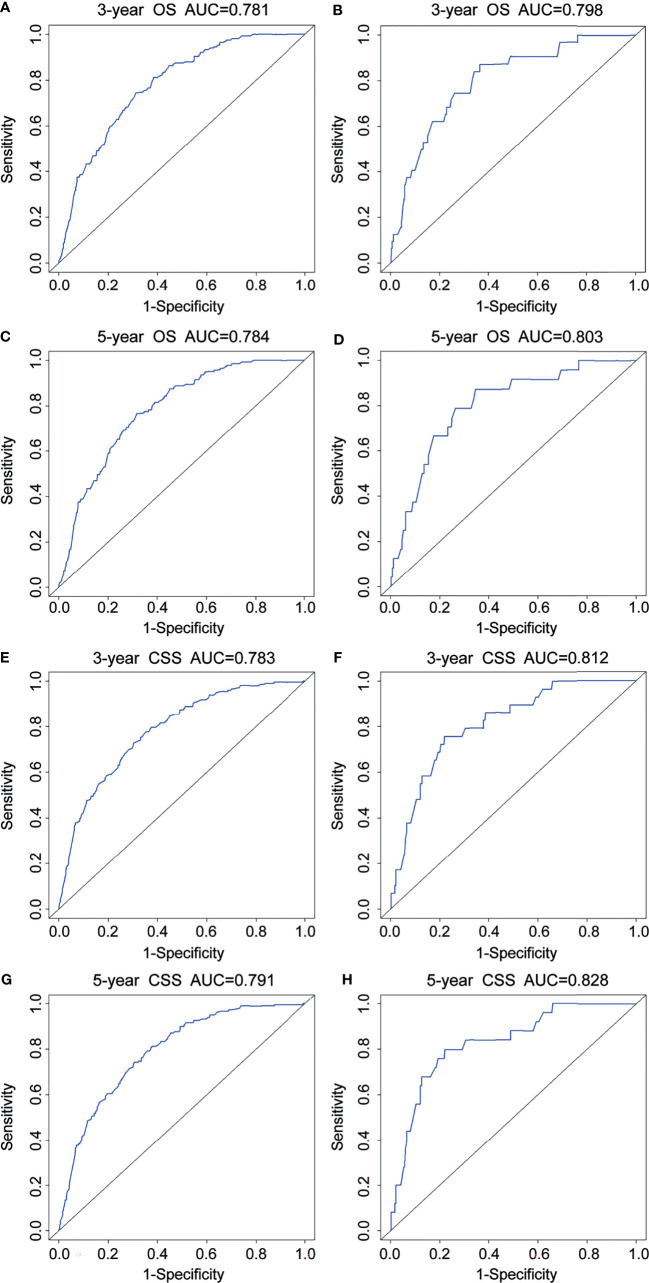
ROC curves for 3- and 5-year OS and CSS of the prognostic nomograms. ROC curves for 3- and 5-year OS in the training cohort **(A, C)** and the validation cohort **(B, D)**. ROC curves for 3- and 5-year CSS in the training cohort **(E, G)** and the validation cohort **(F, H)**. OS, overall survival; CSS, cancer-specific survival.

For the prediction of OS, the prognostic nomogram showed a C-index of 0.788 (95% CI, 0.762–0.814) and 0.777 (95% CI, 0.758–0.796) in the training cohort and the validation cohort, respectively. As for CSS, the C-index of the prognostic nomogram in the training cohort and the validation cohort was 0.792 (95% CI, 0.767–0.817) and 0.781 (95% CI, 0.764–0.798), respectively. Moreover, the calibration plots for prognostic nomograms showed that predictions of the 3- and 5-year survival probability models of OS and CSS were almost keeping with actual observations, whether in the training cohort or the validation cohort (**Figure 4**). Furthermore, as shown in the ROC curves for prognostic nomograms, the 3- and 5-year AUCs for the nomogram of OS were 0.781 and 0.784 in the training cohort (**Figures 5A, C**), and 0.798 and 0.803 in the validation cohort (**Figures 5B, D**), respectively. Meanwhile, the 3- and 5-year AUCs for the nomogram of CSS were 0.783 and 0.791 in the training cohort (**Figures 5E, G**), and 0.812 and 0.828 in the validation cohort (**Figures 5F, H**), respectively. These results indicated that prognostic nomograms demonstrated satisfactory discrimination and excellent predictive accuracy for both OS and CSS prediction.

**Figure 6:**
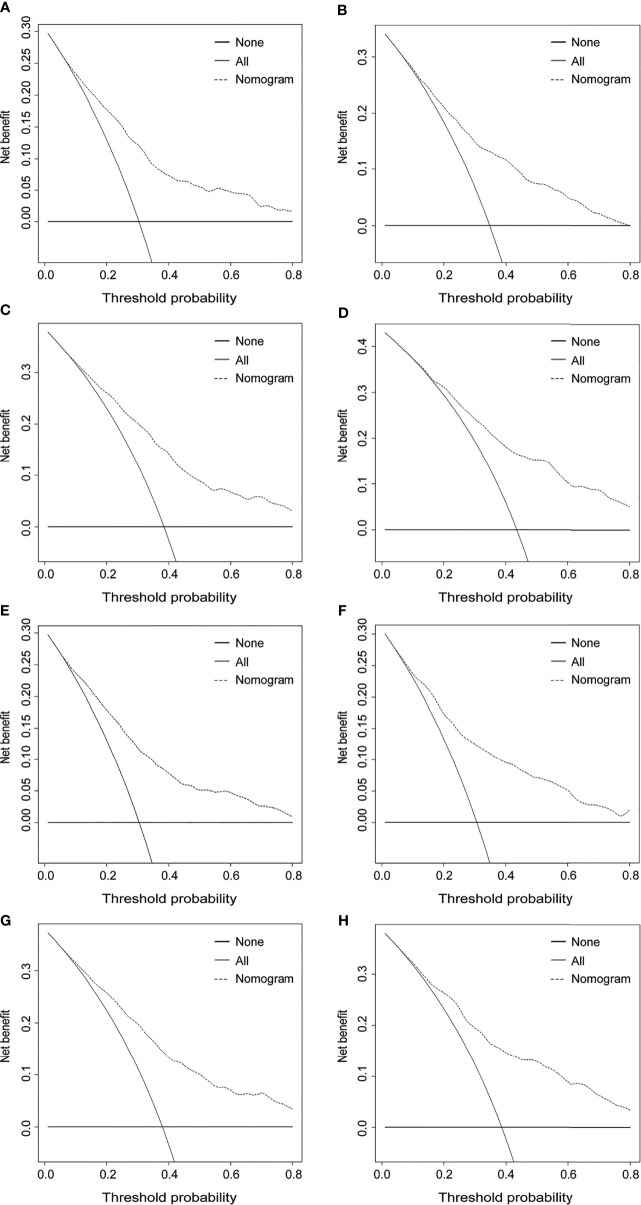
Decision curve analysis for 3- and 5-year OS and CSS of the prognostic nomograms. Decision curves for 3- and 5-year OS in the training cohort **(A, C)** and the validation cohort **(B, D)**. Decision curves for 3- and 5-year CSS in the training cohort **(E, G)** and the validation cohort **(F, H)**. OS, overall survival; CSS, cancer-specific survival.

The DCA was further plotted to estimate the clinical benefits to the patients. The DCA curves illustrated that those nomograms achieved robust positive net clinical benefits across a wide scale of threshold probabilities for the 3- and 5-year OS and CSS prediction, respectively (**Figure 6**). This finding demonstrated that the novel nomograms had remarkable clinical validity in predicting cervical cancer patients with lymph node metastasis.

## Discussion

Cervical cancer is one of the main causes of women’s cancer-related deaths worldwide (15). For cervical cancer patients, lymph nodes status is a critical predictor of survival that has been applied to guide clinical treatment (16). The risk of lymph node metastasis increases per FIGO stage (2009 version), with incidences from 2% (stage IA2) to 14–36% (IB), 38–51% (IIA), and 47% (IIB) in the pelvic region; and from 2% to 5% (stage IB), 10–20% (IIA), 9% (IIB), 13–30% (III), and 50% (IV) in the paraaortic region (17, 18). Once cervical cancer with lymph node metastasis occurs, the survival rate of patients is greatly reduced. The median 5-year survival rate of patients without lymph node metastasis varies between 80% and 100%, whereas for patients with pelvic lymph node metastasis and paraaortic lymph node metastasis, the median 5-year survival rate goes from 57% to 78% and from 47% to 78%, respectively (19, 20). Moreover, Kilic et al. found that the number of positive metastatic lymph nodes may have an effect on survival; the 5-year recurrence-free survival (RFS) was 77% in patients with 5 or fewer positive metastatic lymph nodes, 51% in patients with 6–10 positive metastatic lymph nodes, and 37% in patients with 11 or more positive metastatic lymph nodes (21).

AJCC and FIGO are the two major clinical staging schemes for cervical cancer. Nevertheless, these two clinical stages do not fully reflect the prognosis of cervical cancer patients because of their poor assessment of lymph node status and other important personal factors. Patients in the same clinical stage might have different prognosis outcomes. Therefore, it is necessary to include lymph node status into the discussion of prognostic factors affecting cervical cancer with lymph node metastasis. To this end, we extracted the data of cervical cancer patients with lymph node metastasis from the public SEER database and conducted univariate and multivariate Cox proportional hazard regression analysis to determine the independent prognosis indicators affecting the OS and CSS.

Cox regression analysis revealed that tumor size, LODDS, radiotherapy, surgery, T stage, histology, and grade were identified as significantly independent prognostic variables for OS and CSS. The X-tile software showed that the optimal cutoff points of tumor size were 3.8 and 6.4 cm. Patients with a tumor size between 3.9–6.4 cm and ≥6.5 cm had remarkably lower survival rates than those with tumor size ≤3.8 cm. Moreover, the prognosis of cervical cancer patients with lymph node metastasis noticeably deteriorated as the tumor size increased. Tumor size is a critical prognostic indicator for cervical cancer patients with lymph node metastasis. Horn et al. found that patients with tumor size of ≤2.0 cm had higher OS in the revised FIGO 2018 staging system compared to patients with tumor size of 2.1–4.0 cm and those with ≥4.0 cm (22). Besides, tumor size significantly affects the prognosis of other tumors. Yan et al. reported that tumor size was an independent factor of CSS, RFS, and OS in upper urinary tract urothelial carcinoma after radical nephroureterectomy (23).

Currently, numerous parameters, including the number of positive lymph nodes (NPLN), the ratio of positive to removed lymph nodes (LN ratio, LNR), and LODDS, are applied to assess the status of lymph nodes. Several previous studies have determined that LODDS have a higher prognostic value for survival outcomes than NPLN and LNR. For instance, Yu et al. found that LODDS has a higher linear trend χ^2^ test score, higher likelihood ratio χ^2^ test score, higher Harrell C-index, and lower Akaike information criterion for predicting prognosis of node-positive lung squamous cell carcinoma patients after surgery compared to NPLN and LNR (24). Similarly, LODDS proved to be the best fit for predicting OS and CSS among patients with node-positive non-small cell lung cancer compared with NPLN or LNR (25). Yet, so far, only a few studies have attempted to explore the prognostic value of LODDS in cervical cancer (12, 26), and no studies have reported on its prognostic role in cervical cancer with lymph node metastasis. The results of the X-tile software indicated that the optimal cutoff points of LODDS were -0.9 and -0.2. Patients with LODDS between -0.9 to -0.2 and >-0.2 had remarkably lower survival rates than those with LODDS ≤-0.9. Moreover, Cox regression analysis showed that as the LODDS level increased, the prognosis of cervical cancer patients with lymph node metastasis became worse. Also, a recent study showed that the higher the number of lymph node metastasis, the worse the disease-free survival of patients (27). These results strongly suggest that LODDS can be used as an effective indicator of the survival and prognosis among cervical cancer patients with lymph node metastasis.

Radiotherapy and hysterectomy are recommended treatment options for cervical cancer patients with lymph node metastasis. Lin et al. found superior OS and CSS in FIGO stage I small cell neuroendocrine cervical cancer patients who underwent hysterectomy compared to those who did not undergo surgery; the 5-year OS and CSS for the hysterectomy group were 57.8% and 50.0%, respectively, compared with 29.6% and 27.9% for the nonsurgical group (28). Furthermore, Wu et al. found that patients with stage IB1 and IIA1 cervical cancer who underwent hysterectomy had a longer survival time (29). Huang et al. suggested that the addition of local radiotherapy could lead to better OS and CSS among cervical cancer patients with the M1 stage (30). Similarly, our study found an unfavorable prognosis in cervical cancer patients with lymph node metastasis who did not undergo hysterectomy and radiotherapy. Local radiotherapy can effectively improve the prognosis of cervical cancer patients with lymph node metastasis probably because the metastatic lymph nodes are mostly superficial and clustered. Compared with other treatment options, local radiotherapy can also better control the progression of the disease (31, 32). In patients with advanced cervical cancer, weekly use of cisplatin and volumetric-modulated arc therapy combined with comprehensive, intensive radical therapy significantly improved 3-year survival and local control (33). For the sake of a gratifying prognosis, clinical treatment for cervical cancer patients with lymph node metastasis may favor radical hysterectomy and local radiotherapy. T stage, histology stage, and tumor grade are intrinsic characteristics of tumors that have been proved to be independent prognostic parameters among patients with cervical cancer (34–36).

Based on the multivariate Cox regression analysis results, we attempted to establish and validate novel nomograms for estimating the 3- and 5-year OS and CSS. To the best of our knowledge, this is the first nomogram established for predicting cervical cancer patients with lymph node metastasis. Compared to the AJCC and FIGO staging schemes, more information about patient demographics, tumor characteristics, lymph node status, and treatment options was incorporated in these nomograms, which could minimize the bias caused by personal demographics, tumor heterogeneity, and different treatment options. The C-indexes of the prognostic nomograms for predicting OS and CSS ranged from 0.777 to 0.792, which revealed that our nomograms had satisfactory discrimination ability. The calibration curves for the nomograms fitted well with the 45-degree line, illustrating the consistency between predictions and actual observations for both 3- and 5-year OS and CSS. Moreover, the discriminatory capacity of the prognostic nomograms could be quantified by AUC values. The 3- and 5-year AUCs for the nomogram of OS and CSS ranged from 0.781 to 0.828. Finally, the DCA curves revealed robust positive net benefits under different threshold probabilities. In short, these nomograms provide more practical tools to help clinicians formulate appropriate individualized treatment options for cervical cancer patients with lymph node metastasis, thereby improving the clinical outcomes.

Although the prognostic nomograms were well verified, our study has several limitations. First, as a retrospective study, this research collected data from the SEER database, and patients with missing data for the included factors were excluded, which inevitably led to a selection bias. Second, numerous key items are lacking, especially the chemotherapy regimens, dosage of radiotherapy, and immunotherapy. Only “Yes” or “No” were exhibited in the public database for radiotherapy, resulting in a weakened effect of radiotherapy variables on survival analysis. Third, the data we used to establish and validate the nomograms came from the same database, which imposed certain limitations on the scope of application of our nomograms. After considering these restrictions, further comprehensive verification through multicenter prospective clinical trials is warranted to confirm this estimation.

## Conclusion

In this study, we used the SEER database to identify significant independent prognostic factors that were used to establish novel nomograms for estimating the 3- and 5-year OS and CSS. The validation results indicated that these nomograms have satisfactory predictive performance and may be used as a reliable tool to estimate the prognosis of cervical cancer patients with lymph node metastasis. They may also assist clinicians in formulating desirable personalized treatments and conducting an individual prognostic evaluation.

## Data Availability Statement

The original contributions presented in the study are included in the article/supplementary material. Further inquiries can be directed to the corresponding author.

## Author Contributions

HM designed this research. JY and ZL conducted the statistical analyses and drafted the manuscript. LW, XZ, LP, and CZ extracted the data and processed the figures and tables. All of the authors critically reviewed the manuscript. All authors contributed to the article and approved the submitted version.

## Funding

This work was supported by the 2020 Tianjin Health Science and Technology Project, Science and Technology Talent Cultivation Project (KJ20110), the 2021 Tianjin Health Science and Technology Project, Youth Talent Project (TJWJ2021QN023), and the Tianjin Key Medical Discipline (Specialty) Construction Project.

## Conflict of Interest

The authors declare that the research was conducted in the absence of any commercial or financial relationships that could be construed as a potential conflict of interest.

## Publisher’s Note

All claims expressed in this article are solely those of the authors and do not necessarily represent those of their affiliated organizations, or those of the publisher, the editors and the reviewers. Any product that may be evaluated in this article, or claim that may be made by its manufacturer, is not guaranteed or endorsed by the publisher.
