# Corrigendum: Development and Validation of Novel Nomograms to Predict the Overall Survival and Cancer-Specific Survival of Cervical Cancer Patients With Lymph Node Metastasis

**DOI:** 10.3389/fonc.2022.916310

**Published:** 2022-04-28

**Authors:** Jianying Yi, Zhili Liu, Lu Wang, Xingxin Zhang, Lili Pi, Chunlei Zhou, Hong Mu

**Affiliations:** ^1^ Department of Clinical Laboratory, Tianjin First Central Hospital, School of Medicine, Nankai University, Tianjin, China; ^2^ Department of Clinical Laboratory, The Third Central Hospital, Tianjin, China; ^3^ Tianjin Key Laboratory of Extracorporeal Life Support for Critical Diseases , Tianjin, China; ^4^ Artificial Cell Engineering Technology Research Center, Tianjin, China; ^5^ Tianjin Institute of Hepatobiliary Disease, Tianjin, China; ^6^ Department of Gynecology and Obstetrics, Traditional Chinese Medicine Hospital of Xiaoyi City, Xiaoyi, China; ^7^ Department of Clinical Laboratory, People’s Hospital of Xiaoyi City, Xiaoyi, China

**Keywords:** cervical cancer, lymph node metastasis, nomogram, overall survival, cancer-specific survival

In the published article, there was an error in author information. Jianying Yi should be the sole first author, which means that Zhili Liu is not an equally contributing first author.

The authors apologize for this error and state that this does not change the scientific conclusions of the article in any way. The original article has been updated.

## Publisher’s Note

All claims expressed in this article are solely those of the authors and do not necessarily represent those of their affiliated organizations, or those of the publisher, the editors and the reviewers. Any product that may be evaluated in this article, or claim that may be made by its manufacturer, is not guaranteed or endorsed by the publisher.

